# Flavaspidic acid BB combined with mupirocin improves its anti-bacterial and anti-biofilm activities against *Staphylococcus epidermidis*

**DOI:** 10.1186/s12866-022-02578-y

**Published:** 2022-07-15

**Authors:** Zhiling Cai, Zitong Mo, Shiqian Zheng, Shihua Lan, Shengjun Xie, Jinghui Lu, Chunping Tang, Zhibin Shen

**Affiliations:** 1 School of Traditional Chinese Medicine, Guangdong Pharmaceutical University, Guangzhou, China; 2 The First Affiliated Hospital of Guangdong Pharmaceutical University, Guangzhou, China; 3 Guangdong Provincial Engineering Center of Topical Precise Drug Delivery System, Guangdong Pharmaceutical University, Guangzhou, China; 4 Guangdong Cosmetics Engineering &Technology Research Center, Guangdong Pharmaceutical University, Guangzhou, China

**Keywords:** Combination therapy, Flavaspidic acid BB, Biofilm, *Staphylococcus epidermidis*

## Abstract

**Background:**

The increase in drug-resistant opportunistic pathogenic bacteria, especially of antibiotic-resistant *Staphylococcus epidermidis* (*S. epidermidis*), has led to difficulties in the treatment of skin and soft tissue infections (SSTI). The major reason for bacterial resistance is the formation of bacterial biofilm. Here, we report a promising combination therapy of flavaspidic acid BB (BB) and mupirocin, which can effectively eradicate the biofilm of *S. epidermidis* and eliminate its drug resistance.

**Result:**

The susceptibility test showed that the combination of BB and mupirocin has good antibacterial and antibiofilm activities, and the fractional inhibitory concentration index (FICI) of BB combined with mupirocin was 0.51 ± 0.00 ~ 0.75 ± 0.05, showing synergistic effect. Moreover, the time-kill curve assay results indicated that the combination of drugs can effectively inhibit the planktonic *S. epidermidis*. After drugs treatment, the drug-combination showed significantly inhibitory effects on the metabolic activity and total biomass in each stage of biofilm formation. The synergistic effect is likely related to the adhesion between bacteria, which is confirmed by field emission scanning electron microscope. And the expression level of *aap*, *sarA* and *agrA* genes were detected by real-time quantitative PCR (qRT-PCR).

**Conclusion:**

Our study provides the experimental data for the use of BB for the clinical treatment of skin infections and further demonstrate the potential of BB as a novel biofilm inhibitor.

## Background

SSTI refer to infectious inflammatory diseases that occur after the skin, its accessory organs, and subcutaneous tissues are infected by pathogenic microorganisms. SSTI are very common clinical diseases. The severity of these diseases varies greatly, and they can be mild, superficial, localized infections or life-threatening deep necrotizing soft tissue infections. SSTI can be roughly divided into superficial skin bacterial infections and secondary SSTI and necrotizing soft tissue infections based on the cause, location, and severity of the disease. The clinical management of SSTI is based on the "Practice Guidelines for the Diagnosis and Management of SSTI" published by the Infectious Diseases Society of America in 2014 [1]. According to the results of epidemiological statistical studies on SSTI, *Staphylococcus* is the most common pathogenic bacteria causing SSTI [2–4]. Among them, the detection rates for *Staphylococcus* in descending order are as follows: *Staphylococcus aureus*, *S. epidermidis*, and *Staphylococcus hemolyticus*. With the widespread clinical application of broad-spectrum antimicrobials, drug resistance of opportunistic pathogenic bacteria associated with SSTI has become increasingly serious. Infections caused by coagulase-negative *Staphylococci* represented by *S. epidermidis* are increasing every year [5], while the discovery of strains resistant to mupirocin, erythromycin, chloramphenicol, and fusidic acid, which are used as topical antimicrobial drugs for the treatment of skin and superficial trauma infections, has also been reported [6–8].

The major reason for bacterial resistance is that pathogens can form a bacterial biofilm (BBF), which protects them from host defenses and prevents the release of certain antibiotics [9]. Their structure is characterized by matrix accumulation, can be divided into four stages, mainly the initial adhesion stage, colony aggregation and formation stage, maturation stage, and shedding stage [10]. Gene expression and protein production of bacteria within BBF are different from those of planktonic bacteria [11, 12]. Studies have found that compared with planktonic bacteria, bacteria in the BBF have 10–1000 times higher drug resistance [13]. Therefore, the development of effective, safe, and low-resistance anti-biofilm drugs is urgently required. The formation of biofilm is regulated by a variety of genes and proteins. The accumulation associated protein (Aap) is an adhesion protein that is involved in *ica*-independent biofilms in *S. epidermidis*, encoded by the *aap* gene, which induces aggregation and adhesion between bacteria [14, 15]. When bacteria aggregate to form microcolonies, polysaccharide intercellular adhesion (PIA) plays a key role, and the sarA gene can affect the formation of PIA, thereby controlling the formation of biofilms [16, 17]. Besides, The *agrA* gene is a response regulator of the agr quorum sensing system, which activates the expression of the system and regulates the expression of RNAIII, regulates the spreading of biofilm and shedding. And the *agr* system has been found to enhance biofilm detachment through the up-regulation of the expression of detergent-like peptides [18].

*Dryopteris fragrans* (L.) Schott. belongs to the genus Dryopteris in the Dryopteris family and is often used to treat various skin diseases such as psoriasis, dermatitis, and various rashes. According to pharmacological studies, *D. fragrans* has multiple active functions such as anti-bacteria, anti-allergy, anti-arthritis, anti-tumor, and anti-oxidation properties [19–23]. Its main active ingredients are phloroglucinol compounds (flavaspidic acid BB and AB, isoflavaspidic acid PB, aspidin BB, aspidin PB, dryofragin, aspidinol) [24–27]. Our group have isolated a variety of phloroglucinol compounds from this plant and experiments showed that phloroglucinol compounds have strong antimicrobial activity against pathogenic bacteria and fungi of a variety of infectious skin diseases such as *S. epidermidis*, *S. hemolyticus*, *Trichophyton rubrum*, *Microsporum gypseum*, *Microsporum canis*, *Marassezia fufur*, etc. [28–30]. Moreover, preliminary studies have found that BB has a significant antibacterial and anti-biofilm activities on Gram-positive bacteria such as methicillin-resistant *Staphylococcus aureus* (MRSA), *Enterococcus faecalis* and *Enterococcus faecium* [31].

However, there is no report on the drug susceptibility study of BB combined with antibiotics as well as its effect on biofilms and the corresponding mechanism. Therefore, in this study, the effects of a combination of BB and mupirocin against *S. epidermidis* and its biofilm formation were evaluated. Therefore, to meet the urgent need for clinical medication, based on the preliminary laboratory research, in this study, the inhibitory effects of BB combined with mupirocin on the biofilm of *S. epidermidis* were evaluated. The experimental results show that the combination of the two drugs has synergistic and inhibitory effects on all stages of biofilm formation and can regulate genes related to biofilm formation. In addition, whether the combination can reverse the resistance of bacteria to antibiotics with increased efficacy at a reduced dose was examined. This research provides experimental data and the theoretical basis for the development of BB as a new type of biofilm inhibitor and guidance for the clinical treatment of SSTI.

## Results

### Antibacterial activities on *S. epidermidis*

With 11 clinically isolated *S. epidermidis* as test strains, the two-fold dilution method was used to determine the minimum inhibitory concentration (MIC) of BB and mupirocin against *S. epidermidis*. The micro checkerboard dilution method was used to determine the FICI of BB and mupirocin for *S. epidermidis*.

The results showed that the FICI value range of the combined use of BB and mupirocin was 0.30 ± 0.03—0.48 ± 0.05, both of which were less than 0.5, which could enhance the inhibitory effect on *S. epidermidis* and showed a synergistic effect (Table 1). Notably, SE04 showed resistance to mupirocin, while its MIC value against mupirocin dropped from 5120.00 μg/mL to 666.67 μg/mL after the drug combination, indicating that the combination of BB and mupirocin can effectively improve the susceptibility of SE04 to mupirocin. SE08 was more sensitive to mupirocin, and its MIC value of BB was significantly decreased after the combination, compared to BB used alone. Meanwhile, the FICI of SE04 and SE08 were 0.36, both lower than most of the other tested strains. Therefore, strains SE04 and SE08, which were resistant and sensitive to mupirocin, respectively, and also responded better to the combination, were selected for subsequent experiments.

**Table 1:** The results of the combined susceptibility test of flavaspidic acid BB and mupirocin

Strain No	BB (μg/mL)		mupirocin (μg/mL)		FICI	Effects of actions ^α^
	MIC_single_	MIC_combined_	MIC_single_	MIC_combined_		
SE01	44.67	15.00	20.00	1.56	0.30(± 0.03)	Synergistic effect
SE02	60.00	20.00*	4266.67	453.33**	0.45(± 0.08)	Synergistic effect
SE03	44.89	11.25	0.098	0.015	0.43(± 0.08)	Synergistic effect
SE04	45.91	18.33	5120.00	666.67**	0.36(± 0.08)	Synergistic effect
SE05	48.00	10.00*	5120.00	1280.00**	0.48(± 0.05)	Synergistic effect
SE06	144.00	36.00**	5120.00	768.00**	0.40(± 0.05)	Synergistic effect
SE07	50.00	10.94*	25.00	5.00	0.42(± 0.08)	Synergistic effect
SE08	40.00	4.38*	32.50	6.16	0.36(± 0.03)	Synergistic effect
SE09	25.00	3.75	20.00	4.38	0.41(± 0.10)	Synergistic effect
SE10	41.30	13.75	5120.00	960.00**	0.44(± 0.06)	Synergistic effect
SE11	40.00	12.50	20.00	2.19	0.41(± 0.10)	Synergistic effect

^α^The effect of the combined medication is determined by the FICI value (the standard deviations (SD) of the FICI value is shown in parentheses). * denote significant differences between the groups of drug-combination and single drug, as determined by one-way ANOVA (*means *P* < 0.05, ** means *P* < 0.01)

### Time-kill curve analysis

**Fig. 1:**
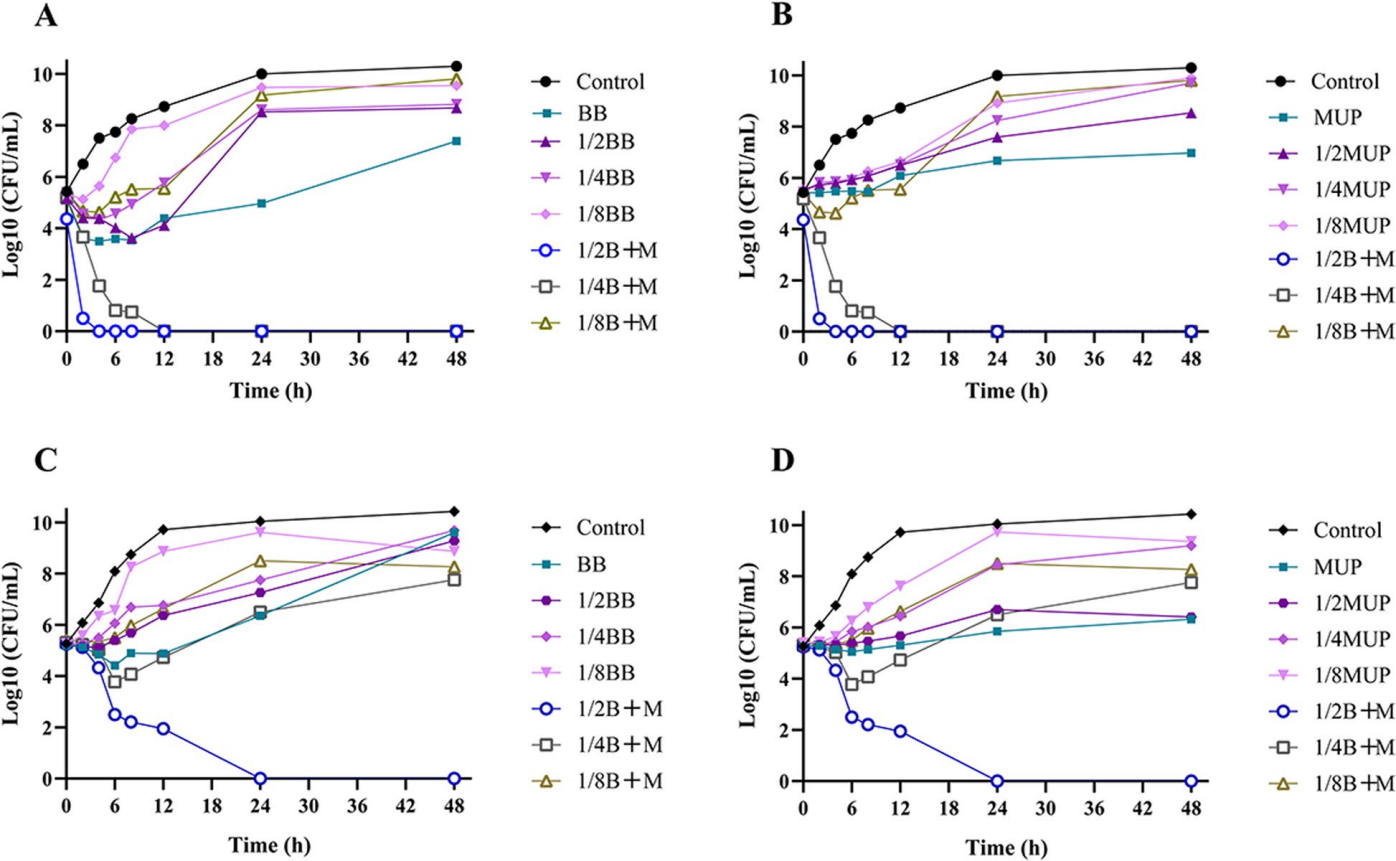
Time-kill curve of *S. epidermidis* under the combination of BB and mupirocin. The viable bacteria were counted at 0, 2, 4, 6, 8, 12, 24, and 48 h of bacterial growth. The ordinate is the colony count, expressed in the form of log10 (CFU/mL). (**A**)-(**B**) SE04; (**C**)-(**D**) SE08. Control: Wells with no drugs; BB: flavaspidic acid BB; MUP: mupirocin; B + M: flavaspidic acid BB + mupirocin; 1/2BB: 1/2 MIC of flavaspidic acid BB; 1/2B + M: 1/2 MIC of flavaspidic acid BB combined with 1/2 MIC of mupirocin, and so on. The control and combination groups in panels A and B, C and D are the same

Time-kill curves are shown in Fig. 1. The kinetic antimicrobial effect of the drug combination on the strain was explored, and the effect of the combined application was further elucidated.

For SE04 and SE08, the BB and mupirocin groups both reduced the viable cells within 48 h, compared with the control group, suggesting they have a certain bacterial growth inhibitory effect, but the effect of the drug gradually weakened with the passage of time.

For SE04, in the combination group, 1/2B + M (referred to 1/2MIC of BB combined with the 1/2MIC of mupirocin, the same below) group reduced the number of viable cell to zero within 4 h, and the 1/4B + M group also reduced the number of viable cell to zero within 12 h. The viable cell quantities in the 1/2B + M and 1/4B + M group were both reduced by > 2 log10 CFU/mL compared with the single-drug group, which showed a good killing effect on SE04. For SE08, the 1/2B + M group reduced the viable cell to zero within 24 h and reduced the the viable cell quantities by ≥ 2 log10 CFU/mL compared to the single-drug group. It is indicating that the synergistic inhibitory effect of the combination of BB and mupirocin on SE04 and SE08 strains.

### Antibiofilm effect on *S. epidermidis*

#### Determination of the minimal biofilm inhibitory concentration (MBIC)

The MBIC of BB and mupirocin on *S. epidermidis* was determined by the micro-checkerboard dilution method to determine the optimal drug concentration combination. The results showed (Table 2) that the best drug intervention combination concentration for strain SE04 and SE08 were 20 μg/mL BB combined with 640 μg/mL mupirocin, and 20 μg/mL BB combined with 5 μg/mL mupirocin, respectively. Notably, compared to mupirocin used alone, the MBICs of mupirocin on SE04 was significantly lower in the combination group, suggesting that the combination of BB and mupirocin can effectively improve the anti-biofilm activity of mupirocin against drug-resistant *S. epidermidis.*

**Table 2:** Minimum biofilm inhibitory concentration (MBIC) of flavaspidic acid BB and mupirocin

Strain No	BB (μg/mL)		mupirocin (μg/mL)	
	MBIC_single_	MBIC_combination_	MBIC_single_	MBIC_combination_
SE04	320.00	20.00	> 2560.00	640.00**
SE08	160.00	20.00	80.00	5.00

^*^ denote significant differences between the groups of drug-combination and single drug, as determined by one-way ANOVA (** means *P* < 0.01)

#### The antibiofilm activity of antimicrobial agents in *S. epidermidis*

Effects of BB and mupirocin on metabolic activity of biofilm

**Fig. 2:**
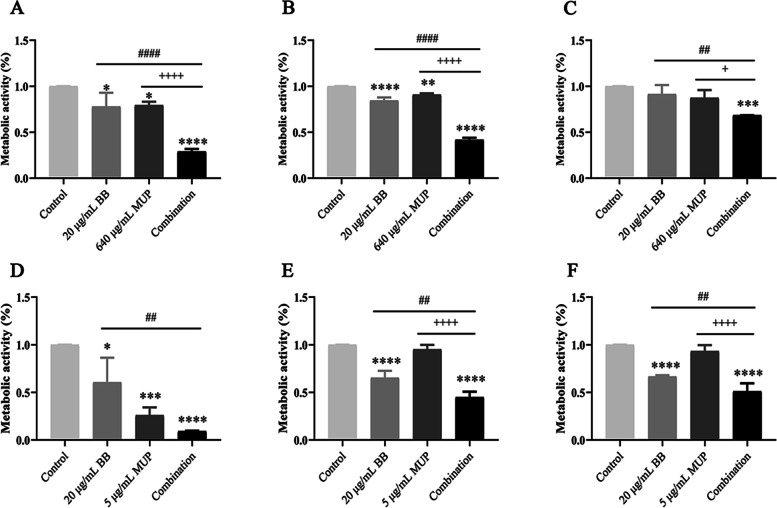
The metabolic activity of the bacteria at each stage of the formation of *S. epidermidis* biofilm. The tested strains were incubated for 6, 24 and 48 h to form adhesion, aggregation and maturity stage of biofilms respectively, and treated with BB and mupirocin, followed by the biofilms being treated with XTT solution, and quantified. **A** SE04 treated for 6 h; **B** SE04 treated for 24 h; **C** SE04 treated for 48 h; **D** SE08 treated for 6 h; **E** SE08 treated for 24 h; **F** SE08 treated for 48 h. Compared with the control group, * means *P* < 0.05, ** means *P* < 0.01, *** means *P* < 0.001, **** means *P* < 0.0001; Compared with the BB group, # means *P* < 0.05, #### means *P* < 0.0001; Compared with the mupirocin group, + means *P* < 0.05, +  +  +  + means *P* < 0.0001

As seen as Fig. 2, after the drug treatment was performed after 6, 24 and 48 h incubation of the tested strains, compared with the control group, the combination groups of SE04 and SE08 can effectively reduce the metabolic activity of bacteria in the biofilm at each stage, and its metabolic activity decreased by 77.1, 58.3, 31.7% (SE04) and 83.8, 55.1, 48.9% (SE08), respectively. For SE04 strain, the inhibition effect of the combination group were better than that of the single-drug groups at each stage. For SE08, compared with BB group, the combination group showed remarkably inhibition of the metabolic activity of the biofilm during the adhesion stage (6 h), and significantly better than the BB and mupirocin groups during the aggregation (24 h) and maturity stages (48 h).

Effects of BB and mupirocin on total biomass quantity of biofilm

**Fig. 3:**
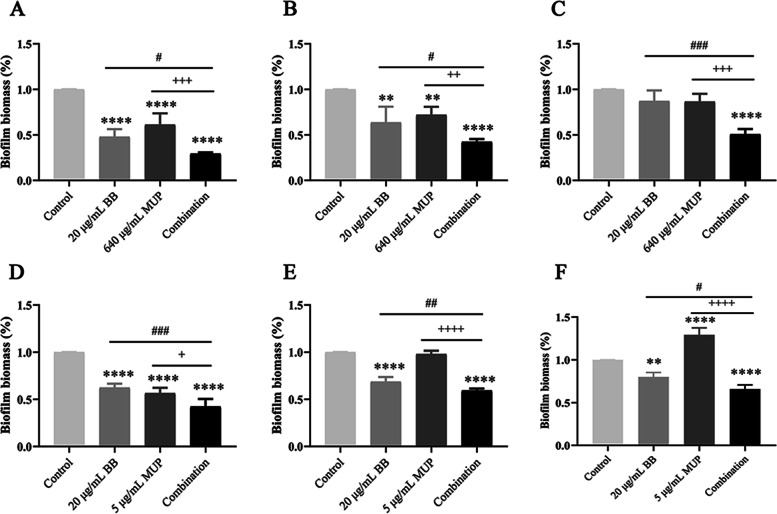
The quantity of biomass at each stage of biofilm formation of the *S. epidermidis*. The tested strains were incubated for 6, 24 and 48 h to form adhesion, aggregation and maturity stage of biofilms respectively, and treated with BB and mupirocin, followed by the biofilms being stained with crystal violet, and quantified. (**A**) SE04 treated for 6 h; (**B**) SE04 treated for 24 h; (**C**) SE04 treated for 48 h; (**D**) SE08 treated for 6 h; (**E**) SE08 treated for 24 h; (**F**) SE08 treated for 48 h. Compared with the control group, ** means *P* < 0.01, **** means *P* < 0.0001; Compared with the BB group, # means *P* < 0.05, ## means *P* < 0.01, ### means *P* < 0.001; Compared with the mupirocin group, + means *P* < 0.05, +  + means *P* < 0.01, +  +  + means *P* < 0.001, +  +  +  + means *P* < 0.0001

After the drug treatment was performed after 6, 24 and 48 h incubation of the tested strains, the results show (Fig. 3) that the combination groups of SE04 and SE08 could effectively inhibit the formation of the biofilm biomass, and the quantity of biomass at each stage was lower than that of the control group (SE04: 70.6%, 57.7%, 49.4%; SE08: 57.3%, 40.7%, 34.2%). At each stage, the quantity of biofilm biomass was significantly decreased in the combination group compared to the single drug groups, indicating that the drug-combination had a significant inhibitory effect on the formed biofilm.

#### FESEM analysis

**Fig. 4:**
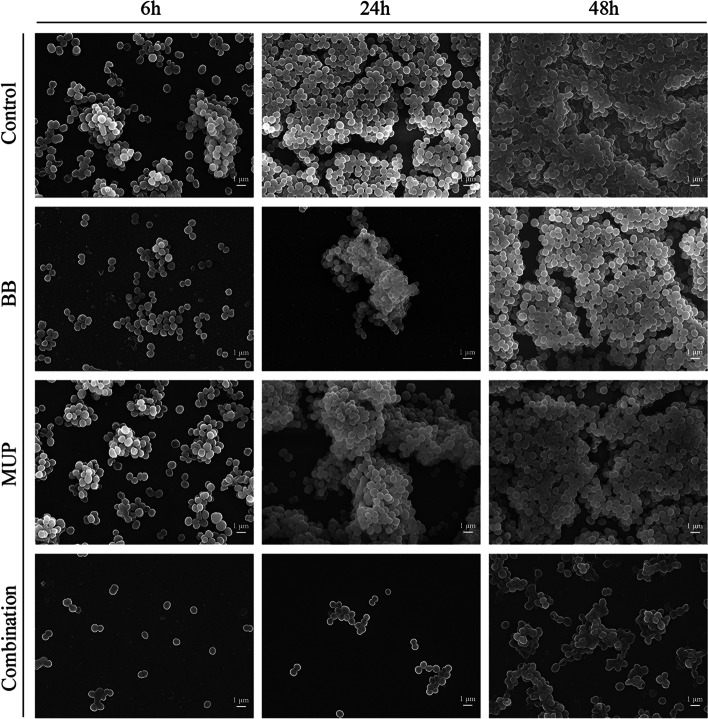
Effect on BB and mupirocin on *S. epidermidis* (SE04) using FESEM analysis. BB: 20 μg/mL flavaspidic acid BB; MUP: 640 μg/mL mupirocin; Combination: 20 μg/mL flavaspidic acid BB + 640 μg/mL mupirocin. Magnification: × 5000

**Fig. 5:**
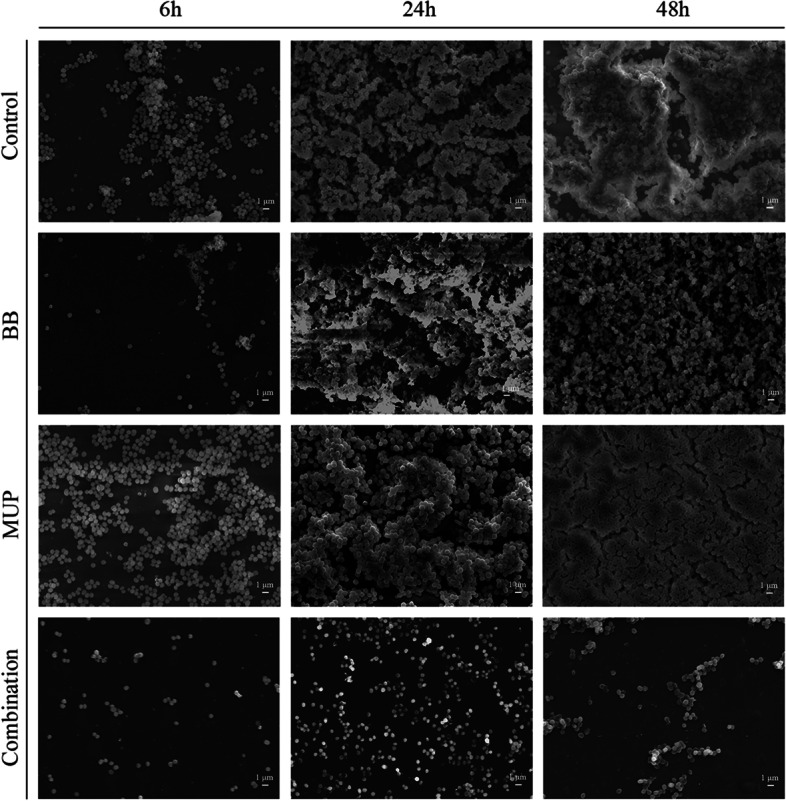
Effect on BB and mupirocin on *S. epidermidis* (SE08) using FESEM analysis. BB: 20 μg/mL flavaspidic acid BB; MUP: 5 μg/mL mupirocin; Combination: 20 μg/mL flavaspidic acid BB + 5 μg/mL mupirocin. Magnification: × 3000

The morphological changes of the in vitro biofilm models of strains SE04 and SE08 at different time periods was observed by scanning electron microscope. The results of FESEM analysis of SE04 (Fig. 4) and SE08 (Fig. 5) are similar.

At adhesion stage (6 h) of biofilm, compared to the control and two single-drug groups, the amount of bacteria in the combination group decreased significantly. This is due to the combination of drugs that increased the antibacterial activity of the drugs and had an early bacterial killing effect.

At aggregation stage (24 h), after the drug treatment, the adhesion between bacteria of combination group was not as tight as that of the control group and each single-drug group, a small amount of cell content exuded, the biofilm structure collapsed.

At maturity stage (48 h), thick and dense mature biofilm have been formed in the control group. Compared to the other groups, in the combination group, the amount of bacteria was significantly reduced, the bacteria did not adhere to each other in sheets but formed clumps, the biofilm structure tended to collapse.

In general, the FESEM observation of the growth trends of the biofilms of strains SE04 and SE08 at different stages are consistent with the above research results.

#### Gene expression analysis of *S. epidermidis*

**Fig. 6:**
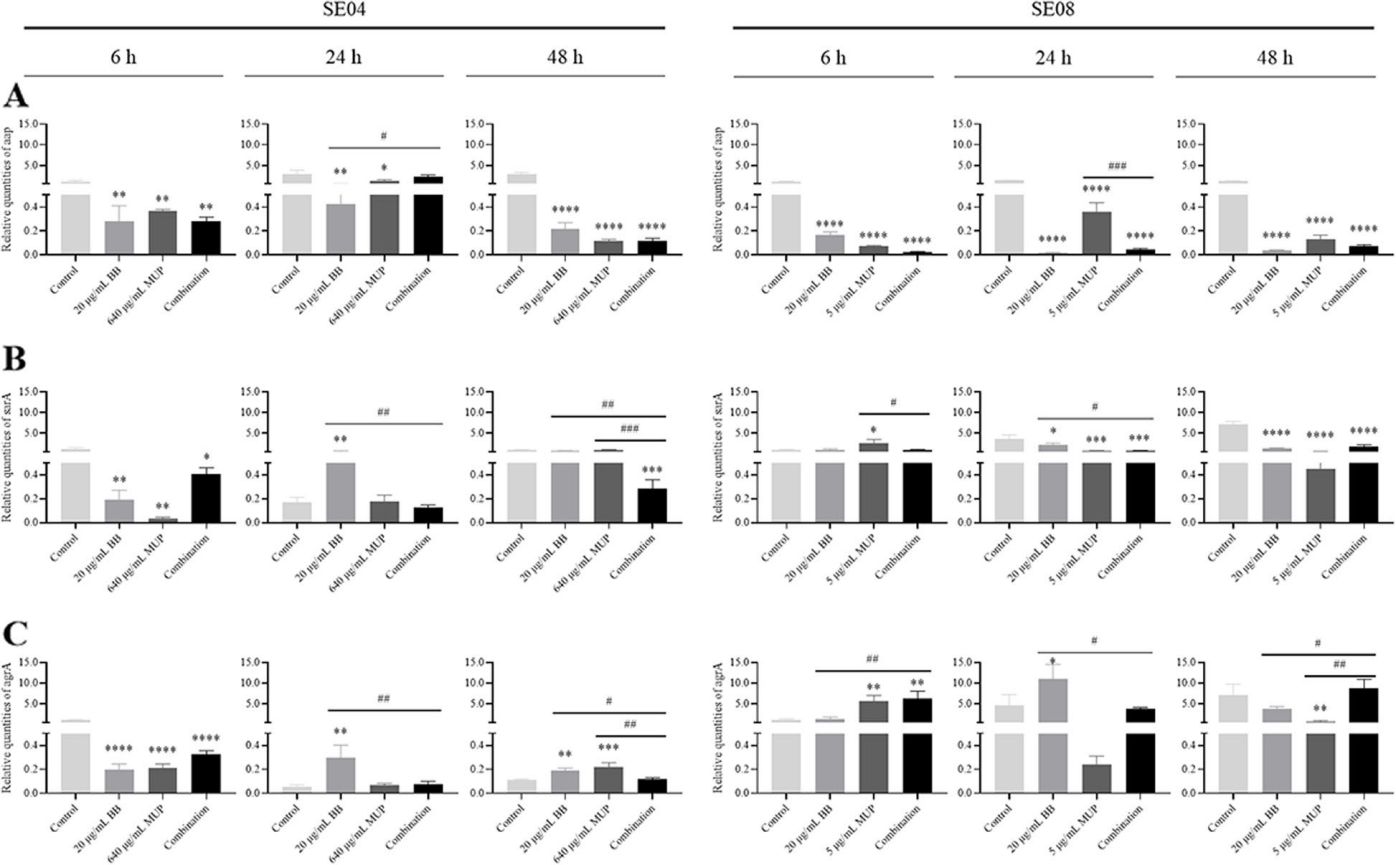
Gene expression analysis of *S. epidermidis*. The SE04 and SE08 were incubated for 6, 24, 48 h to form biofilms, and treated with BB and mupirocin, followed by the biofilms being detected the genes expression by qRT-PCR. **A** The *aap* gene expression level; **B** The *sarA* gene expression level; **C** The *agrA* gene expression level. Compared with the control group, * means *P* < 0.05, ** means *P* < 0.01, *** means *P* < 0.001, **** means *P* < 0.0001; Compared with the BB or mupirocin group, ## means *P* < 0.01, ### means *P* < 0.001

qRT-PCR was used to measure the genes expression levels following the tested drugs treatment. As shown in Fig. 6A, compared to the control group, the biofilms of SE04 and SE08 at all stages showed a reduced expression level of *aap* after the treatment of the drug-combination, among them, the combination group had down-regulated *aap* expression levels in both the adhesion and maturation stages of SE04 biofilms, with the most significant down-regulation in the maturation stage. However, the BB group had lower *aap* expression levels than the combined group in the aggregation stage. Compared with the control group, the combined group significantly down-regulated the *aap* gene expression in all three stages of SE08 biofilm, among which the combination group was significantly better than the mupirocin group in inhibiting the *aap* gene expression in the aggregation stage.

As shown in Fig. 6B, there was a decrease in *sarA* gene expression in the biofilm at all stages of SE04 and SE08 in the combination group after treatment with the drug compared with the control group. The expression level of *sarA* gene was more down-regulated in the combination group at the SE04 aggregation stage compared with the BB group. The combination group had better inhibition of *sarA* gene expression than the single drug group in maturation stages. The *sarA* gene expression was slightly down-regulated in the SE08 adhesion phase in the combination group, and it was significantly more than that in the mupirocin group. The *sarA* gene expression was significantly decreased in the aggregation and maturation phases in the combination group, especially the down-regulation effect in the aggregation phase was significantly better than that in the BB group.

As shown in Fig. 6C, the *agrA* gene expression level decreased after drug treatment in the SE04 biofilm adhesion phase, with the lowest decrease in the combination group. The *agrA* gene expression level increased most significantly in the aggregation phase with BB group. The increase in the maturation phase single drug group was better than the combination group. The expression level of *agrA* gene in the biofilm was up-regulated in all three stages of SE08 after the combination group, and the adhesion stage was better than that of the single drug group. The *agrA* gene expression was most obviously up-regulated in the BB group during the aggregation stage. the single drug group showed down-regulation during the maturation stage, and the *agrA* gene expression was up-regulated in the combination group. This suggested that the combination of BB and mupirocin may regulate the production of virulence factors in cells by inhibiting the expression of *sarA* in *S. epidermidis*. The above results suggested that the combination of of BB and mupirocin could affect biofilm formation by regulating the expression levels of *aap*, *sarA* and *agrA* genes in *S. epidermidis*.

## Discussion

Antibiotic resistance of pathogens has continued to increase, which has severely hindered the treatment of bacterial infections. Specifically, infections caused by *S. epidermidis* have been increasing year by year. Many studies have shown that biofilm is an important factor that affects bacterial resistance [32–34], which has also caused serious clinical problems. Therefore, finding a drug that can effectively inhibit the production of biofilm by bacteria and reduce the drug resistance of pathogens with a view to treating diseases caused by *S. epidermidis* has become a hot topic in research.

In this study, the combination of BB and mupirocin showed a synergistic effect against 11 clinical *S. epidermidis* isolates, including antibiotic resistant and sensitive strains. The time-kill curves were plotted to further confirm the effect that the drug-combination concentration and time-dependent killing had on the antibiotic resistant (SE04) and sensitive (SE08) strains. The time-kill curves clearly showed that, consistent with predictions, the number of *S. epidermidis* viable cells was already below the detection range after sub-MIC BB combined with mupirocin treatment in this study. Accordingly, we suggested that future studies could aim to test the initial doses of these drugs in combination in order to maintain an effective combination therapy throughout the dosing period.

Growth within the biofilm increases the chance of *Staphylococci* protecting themselves from host defenses, antibiotic treatments, and biocides [35]. Therefore, how to effectively prevent the formation of biofilms is still a challenge. This study focuses on a combination therapy regimen that can effectively inhibit the formation of *S. epidermidis* biofilm. Interestingly, the clearance rate of the combination of BB and mupirocin on biofilms shows that it has a inhibitory effect on each stage of the biofilm formation of drug-resistant and sensitive *S. epidermidis*. It can effectively reduce the metabolic activity and the quantity of biomass of biofilm, and the effect is better than that of the each of the single-drug groups. It has been found that phloroglucinol compound extracted from *Callistemon viminalis* exhibit antibacterial activity by disrupting cell membrane structures [36]. In the present study, the results of FESEM analysis showed that a large number of *S. epidermidis* cells exhibited significant depression and rupture after exposure to the combination of BB and mupirocin for 48 h. This demonstrated that the combination of BB and mupirocin bound to the bacterial cell surface and penetrated inside the bacteria, inhibiting bacterial growth. Additionally, previous literature found that aspidin BB, a phloroglucinol compound from *D. fragrans*, induces ROS production by activating NADPH oxidase activity, inhibiting SOD activity and GSH levels, thereby damaging cell membranes, DNA and proteins, and ultimately leading to the death of *S. aureus* [37]. It is suggested that BB may also enhance the antibacterial activity of antibiotics by attenuating the antioxidant defense of cells against antibiotics. More studies are needed to elucidate the mechanism of the interaction between BB and mupirocin.

The *sarA* gene has an important role in regulating the biofilm in the aggregation stage. In addition, it is known that the *sarA* as a central regulatory element, can control the production of virulence factors by *Staphylococcus* [38]. It suggested that the combination may inhibit the production of virulence factors of *S. epidermidis* by suppressing the expression of *sarA*, and inhibit the formation of biofilm. Moreover, previous studies confirmed that subinhibitory concentrations of mupirocin increased the expression level of *sarA* gene in mupirocin-resistant MRSA biofilm [39], which is consistent with the results of this study. In *Staphylococcus epidermidis* the cell wall-anchored protein *Aap* mediates strong adherence to glycan moieties on corneocytes, making this protein seemingly responsible for the permanent colonization of the skin by coagulase-negative *staphylococci* (CoNS) [40]. The results showed that the combination could significantly down-regulate the expression of *aap* gene, suggesting that the combination could inhibite its function of the lectin domain mediates binding to glycan structures of skin cells and achieve inhibition of biofilm formation [14]. Besides, the results of this study showed that the combination showed a down-regulation of the *agrA* in drug-resistant *S. epidermidis* in the maturity stage. It has been found that in the *Staphylococcal* quorum sensing system, when the concentration of AIP (signaling molecule) in the external environment reaches a threshold, *AgrC* undergoes ATP-dependent autophosphorylation and transfers phosphate groups to the response regulator *AgrA*, thereby activating *AgrA*, which then positively regulates the transcription of those genes encoding several extracellular proteases involved in biofilm spreading [18]. Therefore, it is presumed that it may be due to some inhibitory effect of the combination group on the *AgrA/C* two-component signaling system of drug-resistant *S. epidermidis*, which affects the expression of *agrA*, and the exact cause remains to be further confirmed.

In summary, the results of this study showed that BB enhanced the inhibitory effect of antibiotics on biofilm and reduced the antibiotic resistance of *S. epidermidis*. Also, it reduced the dose of antibiotics used. Further studies will focus on the determination of BB cytotoxicity due to safety concerns for clinical application.

## Conclusion

The formation of biofilms of bacteria leads to increased drug resistance, resulting in recurrent or intractable skin and soft tissue infections, caused by *S. epidermidis*. In the present study, the combination of BB and mupirocin show excellent antibacterial and anti-biofilm effects on *S. epidermidis*, which provide experimental data for the use of BB for the treatment of skin infections and further demonstrate the potential of BB as a novel biofilm inhibitor.

## Methods

### Antimicrobial agents and chemicals

Flavaspidic acid BB (BB) was made in the laboratory with a purity of 98%. Mupirocin (80,200,123,218, > 99%) was purchased from Guangzhou Jifan Pharmaceutical Co., Ltd. Cefazolin (190,501, > 98%) was purchased from Guangdong South China Pharmaceutical Group Co., Ltd. Dimethyl sulfoxide (DMSO, Tianjin Baishi Chemical Industry Co., Ltd) was used to prepare a stock solution of BB (64 mg/mL) and mupirocin (256 mg/mL), respectively, stored at 4 °C in the dark. Phosphate buffered solution (PBS) was from Hyclone, USA.

### Bacterial strain and growth conditions

A total of 11 clinically isolated strains of *S. epidermidis* (SE01-SE11), which were donated by Guangdong Lewwin Pharmaceutical Research Institute Co., Ltd., were used. The quality control strain *S. aureus* (ATCC@29,213), was purchased from Guangdong Microbial Culture Collection Center. The bacteria were grown on nutrient agar (NA) medium at 35 °C for 24 h, then inoculated in Caton-adjusted Mueller-Hnton Broth (CAMHB) or tryptone soy broth (TSB) medium (Sigma Aldrich, USA) and diluted to 1 × 10^6^ CFU/mL. During low-temperature storage, all isolates were stored in sterile purified water containing 20% glycerol at -80 °C.

### Antibacterial susceptibility testing

MIC testing was carried out according to the method described by the Clinical and Laboratory Standards Institute [41]. In short, 1 × 10^6^ CFU/mL of the bacterial strain was added to individual wells of a microtiter plate containing 100 μL of CAMHB media and twofold-increasing concentrations of test agent (640- 1.25 μg/mL of BB and 2560- 10 μg/mL of mupirocin). Plates was incubated at 35 °C for 24 h, and the lowest concentration of the agent that inhibits the growth of *S. epidermidis* is considered the MIC.

Fractional inhibitory concentration index (FICI) testing were performed to measure interactions between BB and mupirocin in 96-well plates, as previously described. Briefly, in the checkerboard format, each row of panels contains an decreased concentration of BB (in twofold decrements; 4MIC-1/16MIC), and each column contains an increased concentration of mupirocin (in twofold decrements; 4MIC-1/16MIC). The calculation method of FICI is as follows: (MIC of drug A in combined use/MIC of drug A used alone) + (MIC of drug B in combined use/MIC of drug B used alone) = FICI. FICI ≤ 0.5 denoted synergistic effect; 0.5 < FICI ≤ 1 denoted additive effect; 1 < FICI ≤ 2 denoted irrelevant effect; FICI > 2 denoted antagonistic effect.

#### Quality control

According to the M07-A9 regimen formulated by CLSI [41], the test process and environment need to be tested for quality control during the drug susceptibility test. *S. aureus* (ATCC@29,213) was used as the quality control strain, and cefazolin as the quality control drug. If the MIC of the quality control strain is in the range of 0.25 to 1 μg/mL under the conditions of parallel operation, the determination result is considered valid and reliable.

### Time-kill curve assay

The time-kill curves test was performed based on the method provided by Jiamu Kang [42], with modifications. The strains were treated with different concentrations of BB and mupirocin (MIC, 1/2MIC, 1/4MIC, and 1/8MIC), respectively, and their combination. Tested strains growth were monitored at 0, 2, 4, 6, 8, 12, 24 and 48 h by counting the viable cells on the agar plate at 35 °C. The viable cells were counted after incubation and the rate of decline was determined by plotting the CFU/mL over time. The number of viable cell of the combination group was reduced by ≥ 2 log10 CFU/mL compared with the single-drug group with the best efficacy, which was defined as a synergistic effect [43].

### Antibiofilm activity assay

The MBIC testing were performed to measure the inhibitory activity of the BB or mupirocin on the biofilm [44]. Briefly, a 200 μL of TSB with a microbial concentration of 1 × 10^6^ CFU/mL was filled on 96-well plate and incubated at 35 °C for 24 h. Biofilms were washed to remove nonadherent cells 3 times with PBS, and 200 μL of fresh TSB media was added to each well such that the wells contained increasing concentrations of BB (in twofold increments; 5 to 1280 μg/mL), mupirocin (twofold increments; 5 to 1280 μg/mL), or the combination (twofold increments; 5 to 1280 μg/mL). Plates were incubated for 24 h. The lowest drug concentration without visible turbidity was the MBIC.

### Determination of the inhibitory effect on biofilms

#### Metabolic activity of biofilm assay

XTT testing was used to determine the metabolic activity of *S. epidermidis* biofilm. The method was according to previous research, with modifications [45]. Tested strains suspensions were incubated at a concentration of 1 × 10^6^ CFU/mL in a 96-well plate. After incubating for 6, 24 and 48 h, wells were formed with adherent, aggregated and mature stage biofilms, respectively, the wells were washed to remove nonadherent cells and a new culture broth was added, containing or not different concentrations of drugs (for strain SE04, 20 μg/mL BB, 640 μg/mL mupirocin and their combination; for SE08, 20 μg/mL BB, 5 μg/mL mupirocin and their combination). And incubation was continued for 24 h. An untreated sample was used as a control. Then the wells were washed and dried naturally and 100 μL XTT (0.5 mg/mL)/Vit.K3 (10 mM) mixture reagent was added to each well. After incubating at 35 °C for 2 h in the dark, the bacterial growth (OD450) was measured with a microplate reader (BIO-RAD, USA).

#### Determination of biofilm total biomass

Crystal violet staining test was used to detect the quantity of biomass of biofilm, which was a slight mod-ification of the method reported previously [46]. Preliminary operations are the same as XTT testing. After a new culture broth was added, containing or not different concentrations of drugs and incubating for 24 h. The plates were washed with PBS for 3 times, and methanol (200 μL) was then added to fix the biofilm but was discarded after 30 min. The biofilms were stained with 200 μL of 0.1% crystal violet for 15 min, rinsed with PBS to remove the unadhered dye. Finally, 95% ethanol was added to dissolve the dye, and the biofilm biomass (OD570) was measured with a microplate reader (BIO-RAD, USA).

### FESEM analysis

The FESEM was used to observe the *S. epidermidis* cell morphological changes, according to the previous research [42], with modifications. A 14 mm sterile round slide was placed on a 24-well plate, 1 mL of tested strains suspensions (1 × 10^6^ CFU/mL) was added to each well, which was incubated at 35 °C for 6, 24, and 48 h. The samples were dehydrated in sequentially graded ethanol (30%, 50%, 70%, 80%, 95% and 100%). Dehydrated biofilm samples were sprayed with gold with a vacuum Ion sputtering instrument. The images were obtained using a FESEM (JSM-7610FPlus, JEOL, Japan).

### Gene expression analysis

The *aap*, *sarA* and *agrA* genes expression during the biofilm formation were quantified by measuring the transcript levels of the genes via qRT-PCR. The gene-specific primers were synthesized by Shanghai Sangon Biotechnology Co., Ltd (Table 3). The biofilm at 6, 24, 48 h with or without drug was established according to the previous method. The TRIzol reagent (Invitrogen, USA) was used to extract mRNA, and then the Prime Script RT reagent Kit (RR047A, Takara, Japan) was used to converted to cDNA, following to the manufacturer’s protocol. The reactions were performed with a SYBR Premix EX Taq II Kit (RR820A, Takara, Japan) in accordance with the manufacturer’s instructions. Reactions were performed three times, using 96-well plates. The reaction volume was 20 μL per sample, containing 2 μL of cDNA, 10 μL of SYBR Premix Dimer Eraser, 0.6 μL of 10 μM of each primer, and 6.8 μL of sterile double RNase treated water. The reaction began with a 3 min initial denaturation at 95 °C, followed by 40 amplification cycles of 95 °C for 5 s, 60 °C for 30 s, and 72 °C for 30 s. The relative gene expression was calculated via the 2^− ΔΔCt^ method with 16 s rRNA as the internal reference gene.

**Table 3:** Primer sequences of target genes

Gene ID	Sequence	Product Length (bp)
*aap*-F	5’-TGTCCCATACCCTCTATAGCCTTG-3’	104
*aap*-R	5’-CACCTAGTGCAGCTGGTTTCAG-3’	
*sarA* -F	5’-ATTATTTGCTTCTGTGATACGGTTGT-3’	112
*sarA* -R	5’-ACGTAATGAACACGATGAAAGAACTG-3’	
*agrA*-F	5’-TGTCTTGAAACAGCACATACACGA-3’	97
*agrA*-R	5’-GAACGTATACTGAATTACTTCCCCG-3’	
16S RNA-F	5’-GGCAAGCGTTATCCGGAATT-3’	101
16S RNA-R	5’-GTTTCCAATGACCCTCCACG-3’	

### Statistical analysis

All assays were performed in triplicate. Analyses were performed using GraphPad Prism software version 8.0 (GraphPad Software, Inc., La Jolla, CA). The differences were evaluated with a one-way ANOVA. The differences were considered significant when *p* < 0.05.

## Data Availability

The datasets used and/or analyzed during the current study are available from the corresponding author on reasonable request.

## Abbreviations

*S. **epidermidis*(SE): *Staphylococcus epidermidis*
MRSA: *Methicillin-resistant S. aureus*
BB: Flavaspidic acid BB
MUP: Mupirocin
SSTI: Skin and Soft Tissue Infections
MIC: Minimum Inhibitory Concentration
FICI: Fractional Inhibitory Concentration Idex
MBIC: Minimum Biofilm Inhibitory Concentration
XTT: 2.3-bis (2-methoxy-4-nitro-5-sulfophenyl)-5-[carbonyl (phenylamino)]-2H-tetrazolium hydroxide
FESEM: Field Emission Scanning Electron Microscope
qRT-PCR: Real-time Quantitative Polymerase Chain Reaction
EPS: Extracellular Polymeric Substances
PIA: Polysaccharide Intercellular Adhesion
CLSI: Clinical and Laboratory Standards Institute
ATCC: American Type Culture Collection
NA: Nutrient Agar
CAMBH: Caton-adjusted Mueller-Hnton Broth
TSB: Tryptone soy broth
CFU: Colony-forming unit
DMSO: Dimethyl Sulfoxide
PBS: Phosphate Buffer Saline
